# Cauda Equina Cavernoma: A Case Report and Literature Review

**DOI:** 10.7759/cureus.66586

**Published:** 2024-08-10

**Authors:** João Nogueira, Joana Sobreiro Silva, Leandro Oliveira, Maura Cambango, Pedro Ribeiro

**Affiliations:** 1 Neurosurgery, Hospital de Braga, Braga, PRT; 2 Anatomic Pathology, Hospital de Braga, Braga, PRT

**Keywords:** vascular lesion spine, sciatica pain, intradural extramedular, spine cavernoma, cavernous hemangioma, cauda equina cavernoma

## Abstract

Cavernous hemangiomas (or cavernomas) are benign vasculature malformations that occasionally occur in the central nervous system (CNS). The vast majority is found supratentorial, but cavernomas also appear on the spine, usually intramedullary. Cavernomas in the cauda equina are extremely rare, with only a few cases reported in the literature. We report a case of a cavernoma of the cauda equina in a 69-year-old woman with low back pain and right sciatica for two years. Lumbar MRI showed an intradural mass lesion at the L1-L2 level. She underwent surgery with resection of the lesion, which confirmed the diagnosis of cavernous hemangioma. A good clinical outcome was achieved. In addition to the case report, we present a literature review on all reported cauda equina cavernomas, discussing their clinical presentations, imaging characteristics, histological findings, and surgical management.

## Introduction

Cavernous hemangiomas (or cavernomas) are benign malformations of the vasculature that can occasionally be found in the central nervous system (CNS). They consist of abnormal vascular spaces clustered together without any intervening glial tissue. These lesions are benign [1].

The vast majority of CNS cavernous hemangiomas are located supratentorially [2]. When found, in the spine, they usually develop from the vertebral body and progress by local invasion of the extradural space. Only about 3% of spinal cavernous hemangiomas are localized intradurally, usually intramedullary [3]. Cauda equina cavernomas are extremely rare, with only 39 case reports in the literature.

Here, we report the 40th case involving a 69-year-old woman with a cauda equina cavernoma (around L2).

## Case presentation

**Figure 1:**
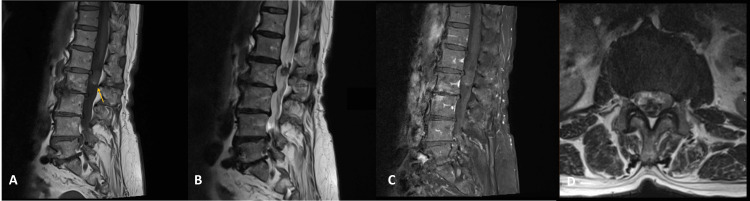
Cauda equina cavernoma on lumbosacral MRI sequences (A) Sagittal T1: An iso-/hyperintense nodular lesion at the L2 level. Arrow: caudal hyperintensity; (B) sagittal T2: hipointense nodular lesion; (C) sagittal post-contrast T1-weighted: almost no contrast enhancement; (D) axial T2

A 69-year-old woman presented with low back pain with right sciatica for two years. She had no motor or sensory deficit in the lower limbs. She underwent a lumbar MRI, which showed an intradural mass lesion with 19 mm in craniocaudal extension at the L1-L2 level, involving the rootlets of the cauda equina. This lesion was hypointense on T1 and T2, with slight hyperintensity in the more caudal component (possibly suggestive of past bleeding) and almost no contrast enhancement (Figure 1).

**Figure 2:**
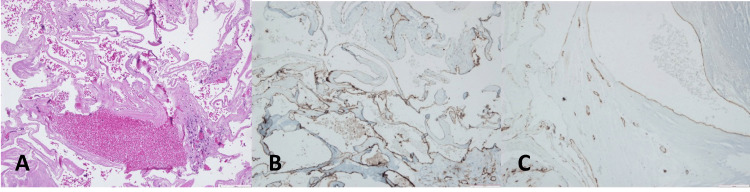
Histological appearance of cavernoma (A) H&E 100x. Large vascular walls with hyaline content; (B) CD31 100X. Endothelial cells staining for CD31; (C) CD34 100X. Endothelial cell staining for CD 34.

The patient underwent surgery, which included a partial L1 and L2 laminectomy. After the dural opening, a mulberry-shaped lesion adhered to a rootlet of the cauda equina was exposed. Using microsurgical techniques, we were able to resect the lesion, preserving the adherent rootlet. The histologic examination showed dilated vessels with hyaline walls and some calcifications compatible with cavernous hemangioma (Figure 2).

**Figure 3:**
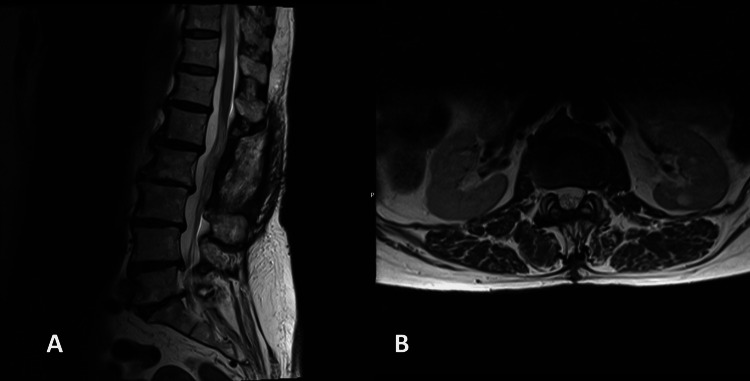
Postoperative MRI showing no recurrence (A) Sagittal T2 and (B) axial T2

After surgery, the lower back pain and sciatica improved, and no neurological deficit was noted. She underwent an MRI two years after the surgery, which showed no recurrence (Figure 3).

## Discussion

Cauda equina cavernomas are extremely rare, being the least frequent site of spinal cavernomas. Since Hirsch et al.'s first case report in 1965 [4], only 39 more have been reported in the literature (Table 1) [1-36].

**Table 1:** Reported cases of cauda equina cavernomas

No	Author, Year	Sex/Age	Level	Presentation	Single/Multiple	Surgery	Miscellaneous
1	Hirsch et al. 1965 [4]	M/20	L2-L3	Motor Deficit; Sphincter Dysfunction	Single	Total+Root Resection	Subarachnoid Hemorrhage
2	Pansini 1966 [5]	M/46	L2	Lower Back Pain; Cauda Equina Syndrome	Single	Total	
3	Ueda et al. 1987 [6]	M/628	L1-L2	Lower Back Pain; Headache	Single	Total	Subarachnoid Hemorrhage
4	Ramos et al. 1990 [7]	F/7	L3	Hakin Adams Triad	Single	Total	
5	Bruni et al. 1994 [8]	M/28	L2	Headache	Single	Total	Subarachnoid Hemorrhage
6	Cervoni et al. 1995 [9]	F/26	L1-L2	Lower Back Pain; Headache	Single	Total	Subarachnoid Hemorrhage
7	Cervoni et al. 1995 [9] ​​​​​​​	M/32	L5	Lower Back Pain; Motor Deficit	Single	Total	
8	Makino et al. 1995 [10] ​​​​​​​	M/67	L2	Headache; Hydrocephalus	Single	Total+Root Resection	
9	Choi et al. 1996 [11] ​​​​​​​	F/46	L1	Lower Back Pain	Single	Total+Root Resection	
10	Rao, 1997 [1]	M/60	L1-L3	Motor Deficit	Single	Total	
11	Duke et al. 1998 [12]	F/49	L4	Lower Back Pain; Radiculopathy	Single	Total	
12	Kim et al. 2001 [13] ​​​​​​​	M/65	L5	Radiculopathy; Motor Deficit	Single	Not mentioned	
13	Park et al. 2003 [14] ​​​​​​​	M/33	L2-L3	Lower Back Pain; Radiculopathy; Headache; Hydrocephalus	Single	Total	Subarachnoid Hemorrhage
14	Jabbour et al. 2004 [15]	M/33	L1-L3	No Symptom	Multiple	Biopsy	Post Radiotherapy
15	Falavigna et al. 2005 [16] ​​​​​​​	F/44	L3-L4	Lower Back Pain; Cauda Equina Syndrome	Single	Total+Root Resection	
16	Chung et al. 2005 [17] ​​​​​​​	M/52	L2	Lower Back Pain; Radiculopathy	Single	Total+Root Resection	
17	Labauge et al. 2006 [18]	M/52	L1-L4	Motor Deficit	Multiple	Biopsy	Post Radiotherapy
18	Caroli et al. 2006 [19]	M/71	L4	Lower Back Pain; Radiculopathy	Single	Total+Root Resection	
19	Miyake et al. 2007 [20]	M/18	L1	Lower Back Pain; Radiculopathy	Single	Total+Root Resection	
20	Cecchi et al. 2007 [21]	F/75	L3-L4	Lower Back Pain; Radiculopathy	Single	Total+Root Resection	
21	Chung et al. 2008 [22]	F/58	L2	Not mentioned	Single	Not mentioned	
22	Chung et al. 2008 [22]	F/59	L4	Not mentioned	Single	Not mentioned	
23	Ducray et al. 2008 [23]	M/42	L5	Motor Deficit	Multiple	Biopsy	Post Radiotherapy
24	Yi et al. 2009 [24]	M/67	L2-L3	Lower Back Pain; Radiculopathy; Motor Deficit	Single	Total	
25	Chun et al. 2010 [25]	F/74	L4	Radiculopathy	Single	Total+Root Resection	
26	Farid et al. 2011 [26]	M/68	L2-L3	Lower Back Pain; Motor Deficit	Multiple	Biopsy	Post Radiotherapy
27	Sulochana et al. 2012 [27]	M/36	L5-S1	Lower Back Pain; Motor Deficit	Single	Not mentioned	
28	Nie et al. 2012 [3]	F/57	L1	Lower Back Pain; Cauda Equina Syndrome	Single	Total	
29	Popescu et al. 2013 [28]	F/60	L4	Lower Back Pain; Radiculopathy	Single	Total	
30	Takeshima et al. 2014 [29]	M/44	L2-L3	Lower Back Pain; Radiculopathy	Multiple (Brain)	Total	
31	Yang et al. 2014 [30]	M/27	L2	Lower Back Pain; Radiculopathy; Sphincter Dysfunction	Single	Total	
32	Yang et al. 2014 [30]	M/59	L3	Lower Back Pain; Radiculopathy	Single	Total+Root Resection	
33	Katoh et al. 2014 [31]	M/36	L1	Headache, Hydrocephalus	Single	NM	Subarachnoid Hemorrhage
34	Mataliotakis et al. 2014 [32]	M/79	L2-L3	Lower Back Pain; Radiculopathy	Single	Total+Root Resection	
35	Kumar et al. 2016 [33]	M/21	L3-L4	Lower Back Pain; Radiculopathy; Motor Deficit	Single	Total	
36	Drazin et al. 2016 [34]	M/76	L2-3	Motor Deficit	Multiple	None	Post Radiotherapy
37	Yaltirik et al. 2016 [35]	F/13	L2	Lower Back Pain; Motor Deficit	Single	Total	
38	Golnari et al. 2017 [36]	M/60	L2	Lower Back Pain; Radiculopathy	Single	Total	Subarachnoid Hemorrhage
39	Apostolakis et al. 2018 [2]	M/77	L3	Lower Back Pain	Single	Total	
40	Nogueira et al. 2024 (current)	F/69	L1-L2	Lower Back Pain; Radiculopathy	Single	Total	

In our review, only cases with tumors starting from the L1 level were included, excluding cases where the tumor originated at the T12 level, even if it had a subsequent caudal extension to lumbar levels.

Cauda equina cavernomas are more common in males than females, with a 2.08 ratio (27/13). The patient's mean age at diagnosis was 50, aged between 13 and 79. Regarding the location, 23 cases were located on a single lumbar level, in descending order: L2 level in eight cases, L4 in five cases, L1 in four instances, and L3 and L5 in three cases each. In 17 cases, the cavernomas extended to two or more levels, the most common location being L2-L3 with seven cases. About 63% (25 cases) were on the upper lumbar spine (L1 and L2).

While most cauda equina cavernomas are solitary, multiple cavernomas were dispersed along the lumbar rootlets in five cases. Those five cases were all reported on patients previously treated with radiotherapy for oncological diseases. In one case, reported by Takeshima et al. [29], the cauda equina cavernoma was found to be associated with multiple brain cavernomas.

Clinically, the majority of cases (35 in total) had some symptom of cauda equina syndrome (sciatic pain, low back pain, motor neuron deficit, and sexual/sphincter dysfunction). Low back pain was the most common symptom (reported in 27 cases), followed by sciatic pain (20 cases), motor deficit (15 cases), and sphincter/sexual dysfunction (five cases). The complete cauda equina syndrome was only reported in three cases. Subarachnoid hemorrhage (SAH) was found in seven cases, five of them with symptoms related to it (headache, vomiting, or nuchal rigidity). Hydrocephalus was a finding in three cases. In only one report, by Katoh, et al. [31], the hydrocephalus was due to SAH. Besides headache, progressive bilateral neurosensorial hearing loss was also a clinical finding in this report, probably related to brain superficial siderosis after the SAH.

Cavernomas are occult lesions on angiography. CT scan is usually not useful for diagnosis as it lacks specificity. MRI is the imaging technique of choice. The typical appearance is a round, reasonably well-defined space-occupying lesion inside the spine canal. They may appear at various intensities on T1- and T2-weighted imaging, depending on the presence of hemosiderin, calcification, and intensity of blood flow. The degree of gadolinium enhancement was variable among cases [20]. On multiple cauda equina post-irradiation cavernomas, the MRI can show multiple small, contrast-enhancing intrathecal lesions involving the cauda equina roots [33]. A hypointense hemosiderin ring on T2-weighted images can be seen in some cases [14]. In our case, the cavernoma was iso- to slightly hyperintense on the T1-weighted image and hypointense on the T2-weighted image, with almost no gadolinium enhancement. On the T1-weighted image, a caudal hyperintensity can be seen, which was interpreted as intratumoral bleeding. Differential diagnoses include schwannomas, ependymomas, meningiomas, medulloblastomas, metastases, lymphomas, astrocytomas, and gangliomas [2].

On histopathological examination, cavernous hemangiomas are characterized as vascular lesions with proliferation of dilated vessels with thickened walls, covered by a single layer of endothelial cells without atypia. These lesions often show the presence of macrophages with hemosiderin pigment, fibrin thrombi, or calcifications. The tumor cells stain strongly for endothelial markers such as CD31, CD34, and ERG, and can stain positive for neuron-specific enolase and S-100 as well [3,16].

Surgery is the treatment of choice for cauda equina cavernomas [19]. Total excision was possible in all cases reported, except in the radio-induced cavernomas of the cauda equina, which only underwent a biopsy. When possible, the nerve root from which the lesion arises is spared with microsurgical technique: only 14 cases reported that this was possible. In 13 cases, the rootlet was resected along with the cavernoma; in five cases, that information was not reported. No new neurological deficit has been reported in cases where the rootlets were resected. Intraoperative neuromonitoring should be used to allow safe resection and decrease the risk of postoperative neurological deficits [20]. No recurrence has been reported in the literature.

## Conclusions

Despite being rare, cauda equina cavernomas should be part of the differential diagnosis of local compression syndrome.

Surgery with total removal is the treatment of choice for these benign lesions, not only to obtain neurological improvement in symptomatic cases but also to obtain a definitive diagnosis and cure the patient, as no recurrence has been reported in the literature.
